# Sequence-based ultra-dense genetic and physical maps reveal structural variations of allopolyploid cotton genomes

**DOI:** 10.1186/s13059-015-0678-1

**Published:** 2015-05-24

**Authors:** Sen Wang, Jiedan Chen, Wenpan Zhang, Yan Hu, Lijing Chang, Lei Fang, Qiong Wang, Fenni Lv, Huaitong Wu, Zhanfeng Si, Shuqi Chen, Caiping Cai, Xiefei Zhu, Baoliang Zhou, Wangzhen Guo, Tianzhen Zhang

**Affiliations:** State Key Laboratory of Crop Genetics and Germplasm Enhancement, Cotton Hybrid R & D Engineering Center (the Ministry of Education), Nanjing Agricultural University, Nanjing, 210095 China

## Abstract

**Background:**

SNPs are the most abundant polymorphism type, and have been explored in many crop genomic studies, including rice and maize. SNP discovery in allotetraploid cotton genomes has lagged behind that of other crops due to their complexity and polyploidy. In this study, genome-wide SNPs are detected systematically using next-generation sequencing and efficient SNP genotyping methods, and used to construct a linkage map and characterize the structural variations in polyploid cotton genomes.

**Results:**

We construct an ultra-dense inter-specific genetic map comprising 4,999,048 SNP loci distributed unevenly in 26 allotetraploid cotton linkage groups and covering 4,042 cM. The map is used to order tetraploid cotton genome scaffolds for accurate assembly of *G. hirsutum* acc. TM-1. Recombination rates and hotspots are identified across the cotton genome by comparing the assembled draft sequence and the genetic map. Using this map, genome rearrangements and centromeric regions are identified in tetraploid cotton by combining information from the publicly-available *G. raimondii* genome with fluorescent *in situ* hybridization analysis.

**Conclusions:**

We report the genotype-by-sequencing method used to identify millions of SNPs between *G. hirsutum* and *G. barbadense*. We construct and use an ultra-dense SNP map to correct sequence mis-assemblies, merge scaffolds into pseudomolecules corresponding to chromosomes, detect genome rearrangements, and identify centromeric regions in allotetraploid cottons. We find that the centromeric retro-element sequence of tetraploid cotton derived from the D subgenome progenitor might have invaded the A subgenome centromeres after allotetrapolyploid formation. This study serves as a valuable genomic resource for genetic research and breeding of cotton.

**Electronic supplementary material:**

The online version of this article (doi:10.1186/s13059-015-0678-1) contains supplementary material, which is available to authorized users.

## Background

Single nucleotide polymorphisms (SNPs) are the most abundant and universal sequence variation across whole genomes, making them attractive markers for the genetic analysis of plants. It is estimated that one SNP occurs every 100 to 300 bp in any genome [1], showing a frequency one magnitude higher than that of simple sequence repeats (SSRs) and other molecular markers [2]. Thus SNP markers are ideal molecular markers for the construction of saturated genetic maps, cloning genes, and quantitative trait loci (QTLs) that control important agronomical traits, marker-assisted crop breeding [3–6], investigating genetic diversity and population structure [7], genome-wide association studies (GWAS) [8], and even to serve as a robust framework for genome assembly [9]. SNP detection was revolutionized by the rapid development of high-throughput next-generation sequencing (NGS) technologies, which generate millions of high quality sequences rapidly and cost-effectively. Through large-scale genome sequencing, high-density haplotype maps containing millions of SNPs have been constructed for rice [10] and maize [11]. However, genome-wide SNP discovery in polyploidy crops is still challenging owing to their large, highly repetitive sequence and three or more subgenomes nature. The large genome size increases the whole genome sequencing cost, and the polyploidization makes the identification of SNPs more complicated, since a large number of homoeologous and paralogous sequences in genomes results in the discovery of many non-allelic SNPs, which are of no use in genetic studies. In order to discover SNPs at a genome-wide level and to reduce the complexity of plant genomes, several techniques, such as complexity reduction of polymorphic sequences (CRoPS) [12], restriction site associated DNA (RAD) [13], genotyping-by-sequencing (GBS) [14], and sequence-based genotyping (SBG) [15], have been used in SNP discovery. Additionally, SNPs within genes can be explored via transcriptome sequencing [5], which has been successfully applied to SNP detection in many species including maize [16], wheat [17], and rape [18]. Nevertheless, these techniques are targeted and based on small areas of genomes, such that the information gained on SNPs cannot cover sequence variation across the whole genome.

In most eukaryotes, the centromere is an essential chromosomal region and usually contains high-copy-number retrotransposons and satellite repeats, which are highly diverse and difficult to map, clone, and sequence. The precise position of the centromere determines the relative positions and linkage relationship between markers in or near the centromere region. Even though the identification of centromeres on genetic maps has been difficult due to highly repetitive sequence and low recombination rate in centromeric region [19, 20], centromeres have been localized to genetic maps in several plants, such as rice [21], maize [22], *Arabidopsis* [23], and *G. raimondii* [24].

Cotton is an important textile and oil crop throughout the world. The cotton species include two commercially important cultivated allotetraploid species, *G. hirsutum* L. and *G. barbadense* L., which have a genome size of 2.5 Gb and large numbers of repetitive sequences derived from an allopolyploidization event between *G. herbaceum* L. (A_1_) and *G. raimondii* (D_5_) Ulbrich approximately 1 to 2 million years ago (MYA) [25]. Due to its complexity, studies on the structure of the cotton genome have lagged behind those of other important crops such as rice and maize. Interspecific crosses between *G. hirsutum* and *G. barbadense* have been widely used to develop inter-specific maps because of the lack of polymorphisms between the intra-species [26–33]. However, these maps are not dense enough to support high-resolution mapping and genetic association studies. It is therefore important to build a well-saturated genetic map to allow deeper genetic research and breeding of this important fiber crop. SNP discovery in cotton was only initiated in recent years, and only a small number of SNP markers have been identified in the cotton genome, therefore few SNP markers were available in existing genetic maps constructed [34–36].

Here, we constructed an ultra-dense genetic map of allotetraploid cotton consisting of nearly 5 million SNPs (Additional file 1) with the largest amount of sequence variation identified in two cultivated allotetraploid cottons to date. This genetic map was used to improve the Upland cotton reference genome assembly, to detect genome rearrangements, and to identify centromeric regions of allotetraploid cotton. Ultimately, this high-precision and high-resolution genetic linkage map will serve as a valuable genomic resource for enhancing our understanding of polyploid genome structure and evolution, and for tagging genome-wide linkage disequilibrium and association studies that target genes to specific functions or traits.

## Results and discussion

### Sequencing and mining of potential SNPs in tetraploid cotton

A total of 59 interspecific F_2_ individuals (THF_2_) were developed from a cross between two cultivated tetraploid cotton species; *G. hirsutum* acc. TM-1 and *G. barbadense* cv. Hai7124. For SNP discovery, two parents were sequenced at relatively high coverage using the Illumina sequencing approach. In total, 154.7 Gb of high quality short read sequences (61.9-fold genome coverage) were generated for TM-1, and 97.9 Gb (39.1-fold genome coverage) for Hai7124 (Table 1). First, an initial draft of the TM-1 genome was obtained from the TM-1 assembly project, which consisted of 17,188 scaffolds (>10 kb, accounting for 92.0 % of all sequence scaffolds). The size of the assembly was 2.2 Gb, with a scaffold N50 size of 210 kb. Further, all high quality reads from TM-1 and Hai7124 were aligned to the scaffolds of TM-1 to allow the detection of putative SNPs using BWA [37] and Samtools [38] softwares. The alignment results showed that 72.5 % and 38.7 % of the short reads (MQ >20) from TM-1 and Hai7124, respectively, were uniquely aligned to the reference genome (Additional file 2), and these locus-specific and high-quality reads were used to detect the interspecific SNPs between TM-1 and Hai7124. Finally, we identified 6,476,899 putative interspecific SNPs, including 5,054,468 simple SNPs, 1,421,857 hemi-SNPs, and 574 complex SNPs, classified as described by Trick [18]. These simple SNPs accounted for nearly 78 % of the total polymorphic SNPs and are potential markers for genotyping of the mapping population.

**Table 1:** Summary of sequencing data from two parents and the mapping population in tetraploid cotton

Sample	Insert size (bp)	Read length (bp)	Raw data (Gbp)		HQ data (Gbp)	
			Total data (Gbp)	Valid sequence depth (×)	Total data (Gbp)	Valid sequence depth (×)
TM-1	180 and 300	2 × 100	185.0	74.0	154.7	61.9
Hai7124	300	2 × 100	111.8	44.7	97.9	39.1
THF_2_^a^	300	2 × 100	938.0	375.2	784.0	313.6

^a^THF_2_: 59 F_2_ populations from a cross between TM-1 and Hai7124. The estimated genome size is 2.5G. The cutoff value for the PHRED quality score for high-quality filtering is 20. The cutoff value for the percentage of read length that should be of given quality is 70

A total genome sequence of 784 Gb was generated for 59 F_2_ individuals; 13.3 Gb for each individual on average, equivalent to 5.3× coverage of the cotton genome (Table 1, Additional file 3). Reads of the 59 F_2_ individuals were mapped against the primary genome sequence and the uniquely mapped reads of high mapping quality (MQ >20) were retained for genotyping. Accurate genotyping of allopolyploid genomes based on short reads with low genome coverage was difficult due to two major obstacles: First, sequencing error and heterozygous regions in the genome led to inaccurate genotyping at given SNP sites, and second, excessive missing genotypes existed due to low genome coverage. To overcome these challenges, we performed a two-step genome-wide genotyping process. First, SNP positions were identified in the initial genotype of 59 F_2_ individuals using a minimum read depth of four and minimum read per allele of one as thresholds. A similarity score was calculated between SNP sites in each scaffold based on the initial genotype, and the SNP sites with high similarity scores were classified into a block in order to avoid mis-assembly in further analysis. Then, a sliding window approach was used to evaluate a group of consecutive SNPs in the same block for genotyping. The minimum read depth in a window containing adjacent SNP sites was 40 to avoid genotyping errors with a single allele at low genome coverage. The genotype of each sliding window was determined based on Bayesian theory (see Materials and Methods). First step screening showed that the missing genotype rate of SNP sites within the initial genotype was 43.95 % and the accuracy of genotype calling at SNP sites for the F_2_ population was 80.07 % (9.87 % A-H miss calls and 10.06 % B-H miss calls; A for homozygous TM-1 genotype, B for homozygous Hai7124 genotype, and H for heterozygous genotype), while in the second step screening, the missing genotype rate of SNP sites was reduced to 5.21 % when the sliding window approach was used and the accuracy of genotype calling at SNP sites was improved to 99.28 %. It is obvious that the accuracy and efficiency of the sequence-based genotyping of the mapping population were greatly improved through the use of the two-step method. As a result, 4,999,048 of 5,054,468 simple SNPs involved in 4,049 recombination bins were genotyped successfully, where a recombination bin represents a class of SNP loci without a recombination node and missing genotype detected. In our study, hemi and complex SNPs detected between two parents were not used for map construction because of their intricate nature that likely represents a mixture of two homoeologous or paralogous sequences due to the sequencing depth of the F_2_ individuals. Modern breeding has enhanced gene flow and post-domestication introgressions through deliberate hybridization between these two species. For example, the reciprocal introgressions between *G. hirsutum* and *G. barbadense* cultivars have developed Acala and Pima cultivar families. Therefore, many of these interspecific SNPs can be used in such introgressions in future cotton breeding.

### Construction of sequence-based ultra-dense genetic and physical maps

**Fig. 1:**
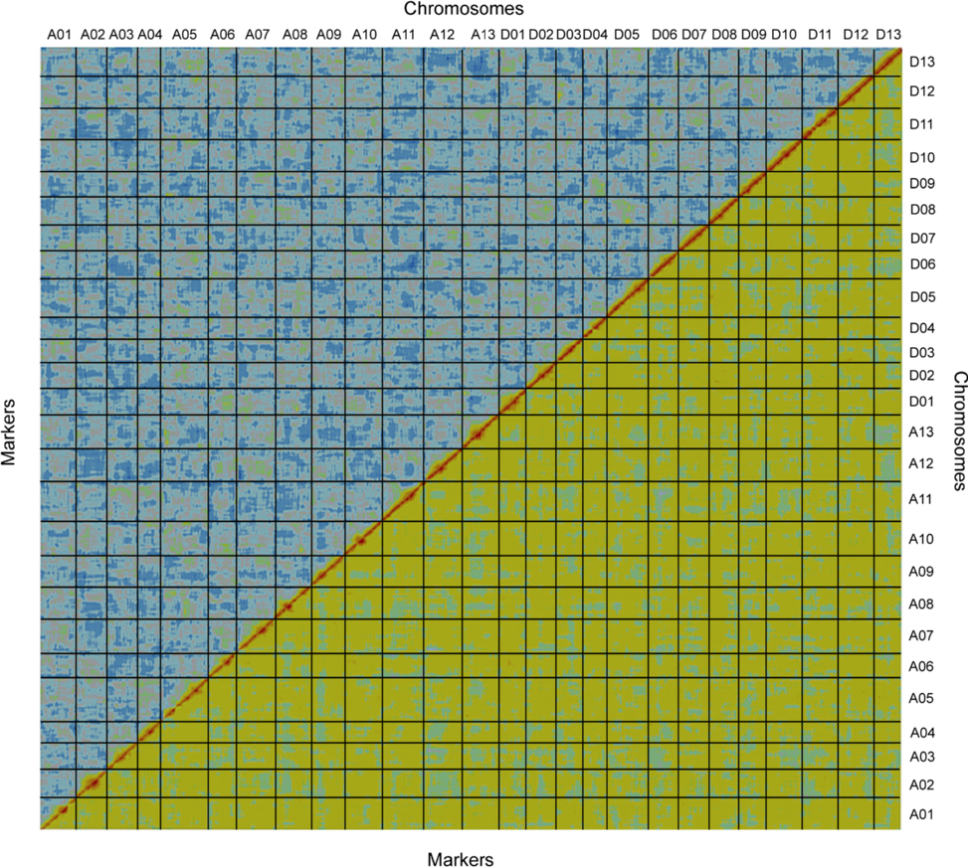
Graphical representation of the high-quality linkage map of tetraploid cotton. Alignment of 4,049 bins along the 26 chromosomes reveals a pairwise BIT score (top-left diagonal) and REC score (low-right diagonal) in all pairs of marker bins, plotted here using CheckMatrix [82]. Red represents tight linkage; yellow represents weak linkage; and blue represents no linkage. The red along the diagonal and the lack of red off the diagonal indicate the high quality and strong statistical support of the map

To effectively order the linkage groups, 441 framework SSR markers that were able to amplify 519 discrete polymorphic loci and were distributed evenly on 26 chromosomes were selected, with 10 cM intervals between the markers based on our microsatellite-based, gene-rich linkage map [33]. The ultra-dense genetic map was constructed based on data from 59 F_2_ individuals and oriented by integrating the reference framework SSRs using Joinmap 3.0 [39]. The map consists of 4,049 recombination bins that include 519 framework SSR loci and 4,999,048 SNP loci, and covers 4,042 cM with an average inter-bin genetic distance of 1.0 cM (Table 2, Additional file 4). Analysis of the SNP calling accuracy and mapping quality using CheckMatrix [10] indicated that all markers in the composite map were assigned to correct linkages in the correct order (Fig. 1). Overall, this SNP array and high-density linkage map is the sole genetic map with the maximum number of SNPs in allotetraploid cotton constructed so far. It will be useful for genomic studies and molecular breeding in cotton.

**Table 2:** Characteristics of the 26 linkage groups in allotetraploid cotton

Linkage group	No. bins	No. SSRs	cM^a^	Inter-bin average distance (cM)	No. scaffolds	Size (Mb)	No. SNPs
A01	157	13	179	1.14	240	115.9	169,307
A02	125	13	122	0.98	368	110.8	245,157
A03	150	19	146	0.98	326	116.7	138,849
A04	119	19	115	0.97	333	93.0	194,383
A05	213	32	213	1.00	313	113.2	136,212
A06	144	24	154	1.07	448	132.3	295,265
A07	163	13	151	0.93	376	101.1	315,158
A08	162	18	168	1.04	444	130.9	294,517
A09	153	28	151	0.99	233	87.0	117,361
A10	175	15	163	0.93	362	121.8	255,072
A11	202	28	205	1.01	407	126.3	260,274
A12	165	16	160	0.97	431	112.6	371,191
A13	168	17	157	0.94	354	115.8	350,161
A subgenome	2,096	255	2,087	1.00	4,635	1477.4	3,142,907
D01	136	27	125	0.92	107	64.2	138,376
D02	143	19	128	0.90	124	71.1	245,387
D03	121	18	128	1.06	101	55.4	182,200
D04	111	12	118	1.06	75	57.1	77,329
D05	213	30	214	1.01	145	66.0	133,959
D06	141	24	145	1.03	117	67.4	208,772
D07	145	16	145	1.00	116	58.4	128,679
D08	144	18	136	0.95	115	69.4	144,574
D09	146	25	157	1.08	122	53.8	116,224
D10	167	18	156	0.94	142	68.2	149,762
D11	174	21	176	1.01	130	72.6	152,575
D12	169	17	174	1.03	121	63.0	94,876
D13	143	19	153	1.07	96	64.5	83,428
D subgenome	1,953	264	1,956	1.00	1,511	831.1	1,856,141
Total	4,049	519	4,043	1.00	6,146	2308.5	4,999,048

^a^Genetic distance was calculated by JoinMap 3.0 [39] using the maximum likelihood mapping method

The inter-specific map was further compared to a high density intra-specific map which was developed to tag QTLs in Upland cotton using RAD sequencing technology [40]. We identified 21,109 SNPs between Upland cotton cultivars, Prema and 86–1, and used these for the genotyping of 161 recombinant inbred lines. Prema is an Acala cultivar which has introgressed segments from *G. thurberi* and *G. barbadense*. Finally, the highest density intra-specific linkage map comprising 4,153 loci over 3500-cM has been developed to date in Upland cotton [40]. The SNP alignments revealed their high consistency in marker order between these two maps (Additional file 5), suggesting the high degree of collinearity between these two cultivated tetraploid cottons.

### Assessing and validating the genomes of tetraploid cottons

Although the emergence of NGS technologies provides a rapid and inexpensive method for genome-wide assembly, the assembly of complex and polyploid genomes such as cotton is still challenging. It is difficult to distinguish homoeologous genomic segments with a high degree of sequence similarity, large gene families, and extensive segmental duplications such as multiple ‘paralogs’. In the present research, the ultra-dense SNP map was further used to validate the initial assembly of the TM-1 genome (scaffold N50: 210 kb) based on the Illumina short reads. We detected 90 instances of mis-assemblies of the scaffolds, which covered 36.0 Mb and accounted for 1.6 % of the entire assembled genome (Additional file 6).

The population size is relatively small for use in resolving smaller scaffolds. Therefore, in addition to the paired-end (PE) reads of 2, 5, and 10 kb libraries used to generate the assembly, the longer scaffolds were enhanced by assembling 174,454 pairs of Sanger-sequenced bacterial artificial chromosome (BAC)-end sequences comprising 116.5 megabases (Mb) [41]. After the BAC-end sequences were merged into the validated assembly, we generated an updated version, V1.0, in which the length of the scaffolds was increased (N50: 1.9 Mb). In the V1.0 assembly, 128 misassembled scaffolds (406.2 Mb, accounting for 16.2 % of the assembly) were detected (Additional file 6). Through comparing the mis-assembled types in the different assembly versions, we found that about half of the scaffold mis-assemblies resulted from homoeologous chromosomes (46.9 % to 52.1 %). More interchromosomal mis-assemblies were detected in the A subgenome chromosomes (21.9 % to 29.1 %) than in the D subgenome, most likely due to the greater number of repetitive sequences in the A subgenome.

After these false mis-assemblies were broken, the final assembly of the TM-1 genome was produced (V1.1, PRJNA248163) [41]. According to the ultra-dense SNP map, which contains 4,999,048 SNP loci, the scaffolds that were >500 kb in length were anchored, and the percentage of anchored scaffolds gradually decreased as the length of the scaffold decreased. Finally, 6,146 scaffolds (covering 2.3 Gb) were anchored and concatenated into 26 pseudo-chromosomes, accounting for 94.9 % (2.4 Gb) of the assembled sequence; 1,477 Mb (4,635 scaffolds) in the A subgenome and 831 Mb (1,511 scaffolds) in the D subgenome. The size of the 26 pseudo-chromosomes varied from 55.4 Mb (D03) to 132.3 Mb (A06) [41].

Allopolyploid plants often undergo major changes in genome structure and function induced by the combination of divergent genomes from two or more related parental species [42]. Many studies have revealed that inter-genomic chromosomal rearrangements, differential gene loss (loss of some duplicates but not others), gene conversion, divergence, and functional diversification of duplicated genes often arise with the onset of polyploidization [43]. Therefore, several assistant strategies such as genetic mapping [3], physical mapping, fluorescent *in situ* hybridization (FISH) [44], and flow-sorting [45] technology, have been employed to aid in whole genome assembly. For example, a chromosome-based draft sequence of the hexaploid bread wheat (*Triticum aestivum*) genome has been produced by sequencing isolated chromosome arms through flow-sorting technology [45]. However, for most polyploidy species with small chromosomes that are difficult to classify and isolate using flow cytometry and sorting, this method is impracticable. In our method, an ultra-dense genetic linkage map is tremendously beneficial in the assembly of large and complex polyploidy genomes; as emphasized by Lewin, ‘every genome sequence needs a good map’ [46]. In fact, the production of high-quality genome assemblies in many species has benefited appreciably from good maps.

### Structural variations in allotetraploid cotton genomes

**Fig. 2:**
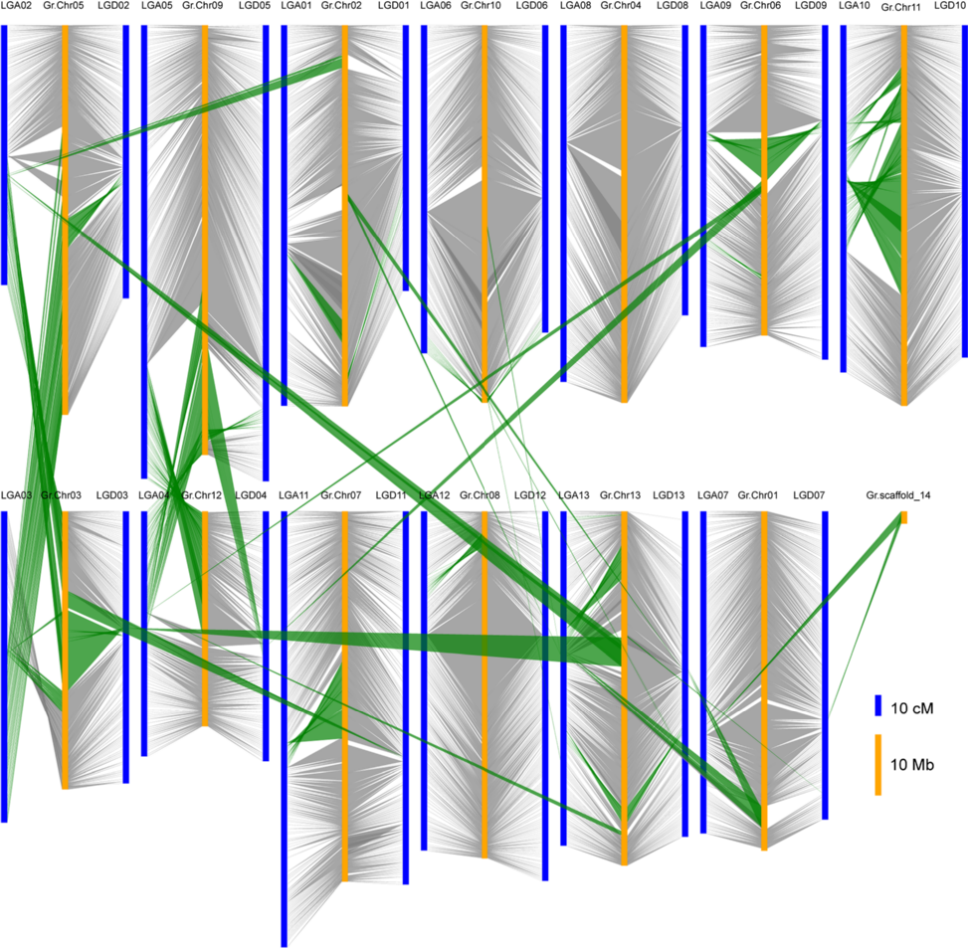
Comparison of the genetic map of tetraploid cotton and the corresponding physical locations on the pseudo molecules of the D genome sequence of *G. raimondii*. TM-1 scaffolds in the 26 linkage groups (blue) were aligned to the D genome sequence of *G. raimondii* (yellow). Genome variations (green blocks) were identified

Generally, ancestors of the A genome species, *G. herbaceum* or *G. arboreum*, and the D genome species, *G. raimondii*, contributed the constituent A and D subgenomes of tetraploid cotton, respectively [25]. Using two sequenced diploid genomes, *G. arboreum* (A_2_) [47] and *G. raimondii* (D_5_) [48], two extant progenitor relatives of tetraploid cotton, the integration of the molecular genetic map with the physical map can be realized. However, this collinearity was not obvious within the A-progenitor genome [47], partly due to numerous examples of mis-assemblies, and partly because *G. arboreum* is an important cultivated diploid species and may have undergone some of its own chromosomal rearrangements during its evolution and improvement. When the ultra-dense tetraploid genetic map was aligned with the *G. raimondii* genome [48], we found a high degree of collinearity, especially in the D subgenome (Fig. 2). Therefore, only the *G. raimondii* genome was used for comparative genomic analysis.

Although the comparative genomic results showed a high degree of collinearity between the genetic map and the physical map, some instances of chromosomal rearrangement were detected between the A, D subgenome and the D genome. Two postpolyploidization reciprocal translocations were displayed between A02 and A03, and between A04 and A05, in the At subgenome (Fig. 2),which broke the chromosomes as follows; A02/A03 (61.90-122.31 cM), corresponding to pseudo molecule Chr03 in *G. raimondii* (GrChr03) at 33736–28298512 bp (28.26 Mb, accounting for 61.75 % of GrChr03), and A03/A02 (66.75-146.42 cM) corresponding to GrChr05 at 17656841–64067579 bp (46.41 Mb, accounting for 72.36 % of GrChr05); A04/A05 (0–54.66 cM) corresponding to GrChr09 at 43817852–70655127 bp (26.84 Mb, accounting for 37.96 % of GrChr09), and A05/A04 (160.43-213.26 cM) corresponding to GrChr12 at 591184–15299554 bp (14.71 Mb, accounting for 41.51 % of GrChr12) (Additional file 7). Furthermore, we identified 15 simple translocations between the A or D subgenome and the D genome, for example, D11 had a simple translocation of 2.75 Mb from GrChr03 (accounting for 6.01 % of GrChr03 at 13111322–15861215 bp). The A and D subgenomes displayed 19 possible inversions that were slightly different from those found by Wang *et al.* [32] and Rong *et al.* [27]. These 19 inversions, with sizes in the range of 0.05 Mb to 37.57 Mb, were found in 16 chromosomes, accounting for 0.09 % to 59.93 % of the corresponding chromosomes. Detailed information on these chromosomal rearrangement events with their locations on both the genetic map and the physical map of *G. raimondii* are presented in Additional file 7. From our comparison of the genetic and physical maps of the D genome sequence, we detected some structural variations not only in the A subgenome, but also in the D subgenome. However, we were unable to determine whether these structural changes occurred in the tetraploid after polyploidization and domestication or in the diploids in the last 1 to 2 million years, since the exact donor species that led to the formation of the tetraploid cotton species 1 to 2 MYA no longer exist.

Two reciprocal translocations revealed in the present study are in agreement with the results from prior studies [27, 32]. Three of 19 possible inversions were almost in agreement with the results of Wang *et al.* [32] and 15 simple translocations are first reported in the study, although we cannot exclude the possibility that some of the newly observed chromosomal structural rearrangements resulted from misassemblies in the *G. raimondii* reference genome sequence. Furthermore, the complex chromosomal rearrangements (inversions and simple translocations) observed here need to be accounted for in the ‘translation’ of information from the D genome in *G. raimondii* to allotetraploid cotton genomes. Genome variations such as inversions and translocations are considered important factors in species evolution. In the process of evolution from diploid to tetraploid cotton, these chromosomal structural rearrangements may have caused a series of changes in phenotype. These structural variations in the genome may help to elucidate the relationships between the phenotype and genotype of important agronomic traits in tetraploid cotton.

### Comparison of the genetic and physical maps of allotetraploid cotton

**Fig. 3:**
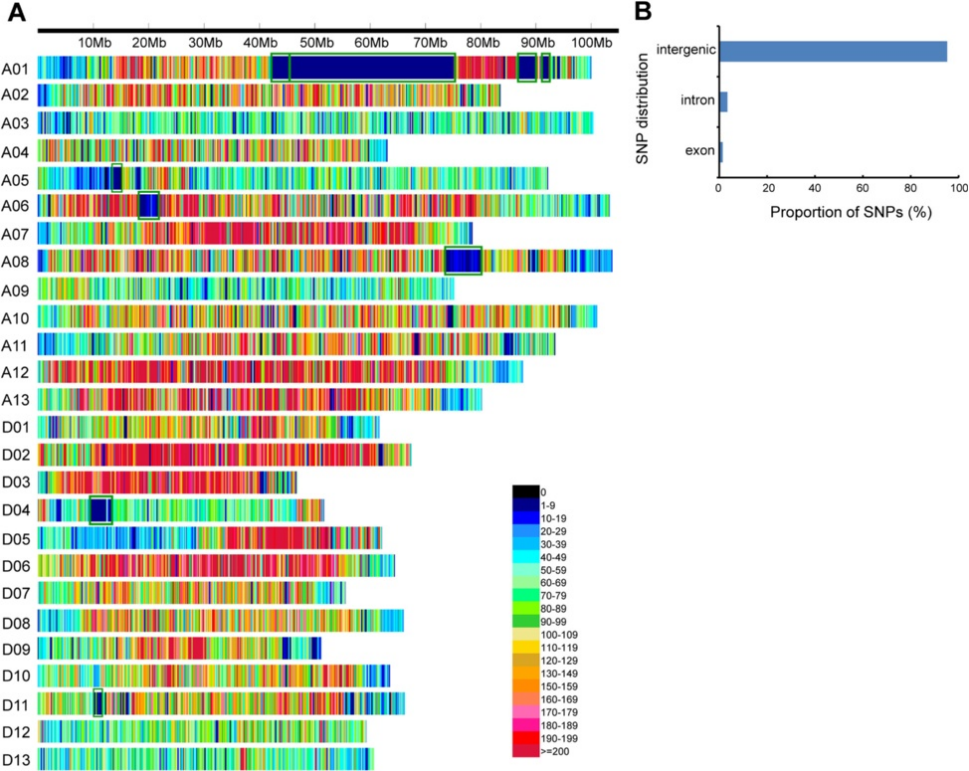
SNP distribution in the cotton genome. **a** Distribution of SNPs in the 26 chromosomes. The x-axis represents the physical distance along each chromosome, split into 50 kb windows. The green rectangles indicate SNP-poor regions. **b** Proportion of SNPs found in intergenic regions, introns, and exons

A total of 4,999,048 mapped SNP loci (1 SNP/0.5 kb) were unevenly distributed on 26 chromosomes, with more loci in the A subgenome (3,142,907) than the D subgenome (1,856,141) (Fig. 3a). On average, each chromosome had 192,271 SNP loci, ranging from as high as 371,191 SNPs on A12, to as low as 77,329 on D04. To explore the patterns of SNP distribution in TM-1 and Hai7124, SNP frequency was plotted at 50 kb intervals along each pseudomolecule (Fig. 3a). Nine SNP-poor regions (at least 20 contiguous intervals with low SNP frequency) comprising a total of 54.0 Mb and accounting for 2.3 % of the whole genome were detected on chromosomes A01, A05, A06, A08, D04, and D11 (Additional file 8). The SNP-poor regions may have been due to historical introgression between *G. hirsutum* and *G. barbadense*. In chromosome A01, for example, some QTLs related to fiber quality from interspecific hybrid populations have been mapped to the SNP-poor region [49–51]. Several investigators have also postulated that an introgression event was responsible for the creation of the Sea Island germplasm [52, 53] in which the introgressed segments play a role in conferring beneficial traits such as high fiber quality or photoperiod neutrality [54]. Furthermore, modern breeding has enhanced gene flow and post-domestication introgressions through deliberate hybridization of these two species. For example, the reciprocal introgressions between *G. hirsutum* and *G. barbadense* cultivars have developed the Acala and Pima cultivar families [55]. Moreover, the vast majority of the mapped SNPs (95.2 %) were located in intergenic regions, and smaller portions were found in intron, followed by exon, regions (Fig. 3b). This suggests that SNP discovery via transcriptome sequencing is limited.

**Fig. 4:**
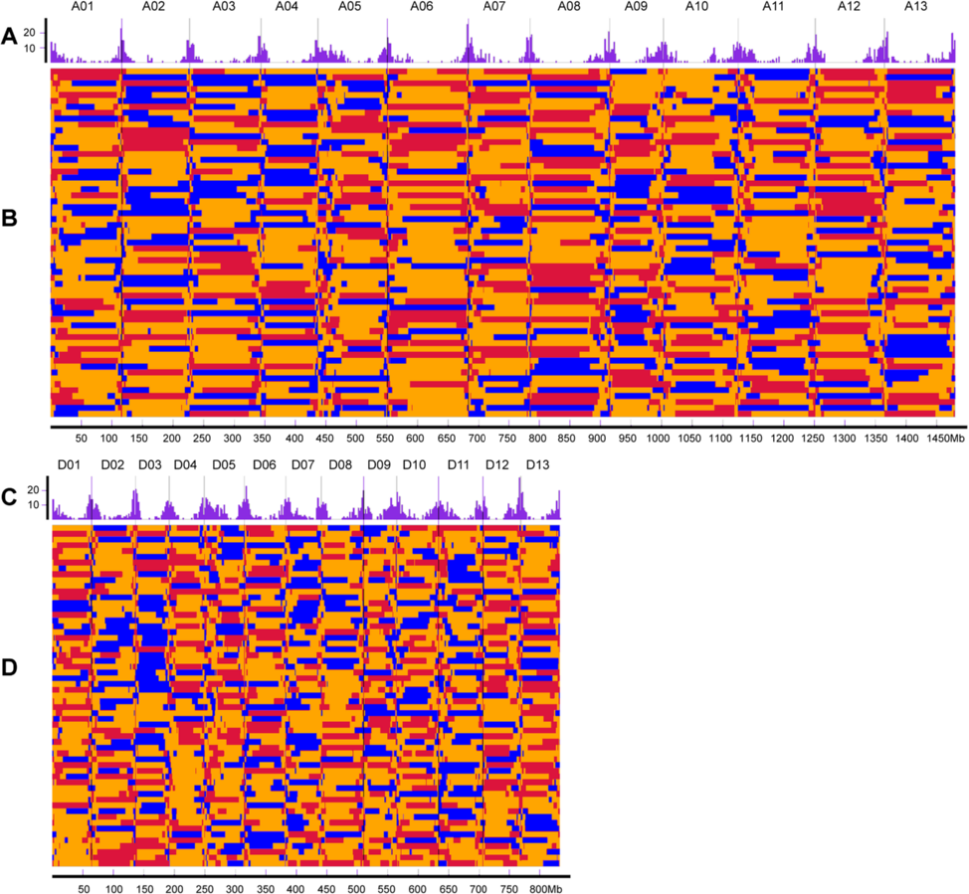
Recombination and bin map for 59 scored F_2_ individuals. **a, c** Frequency distribution of recombination nodes (RNs) per 2 Mb. The vertical scale indicates the number of recombination nodes (RNs); **b, d** Bin maps for the 59 scored F_2_ individual lines. Colored tracks represent the 59 individual lines of the THF_2_ population that were used for linkage map construction: red, alleles inherited from maternal parent (TM-1); blue, alleles inherited from paternal parent (Hai7124); yellow, alleles inherited from heterozygous genotype (TM-1 × Hai7124)F_1_. The horizontal scale indicates physical distance

**Fig. 5:**
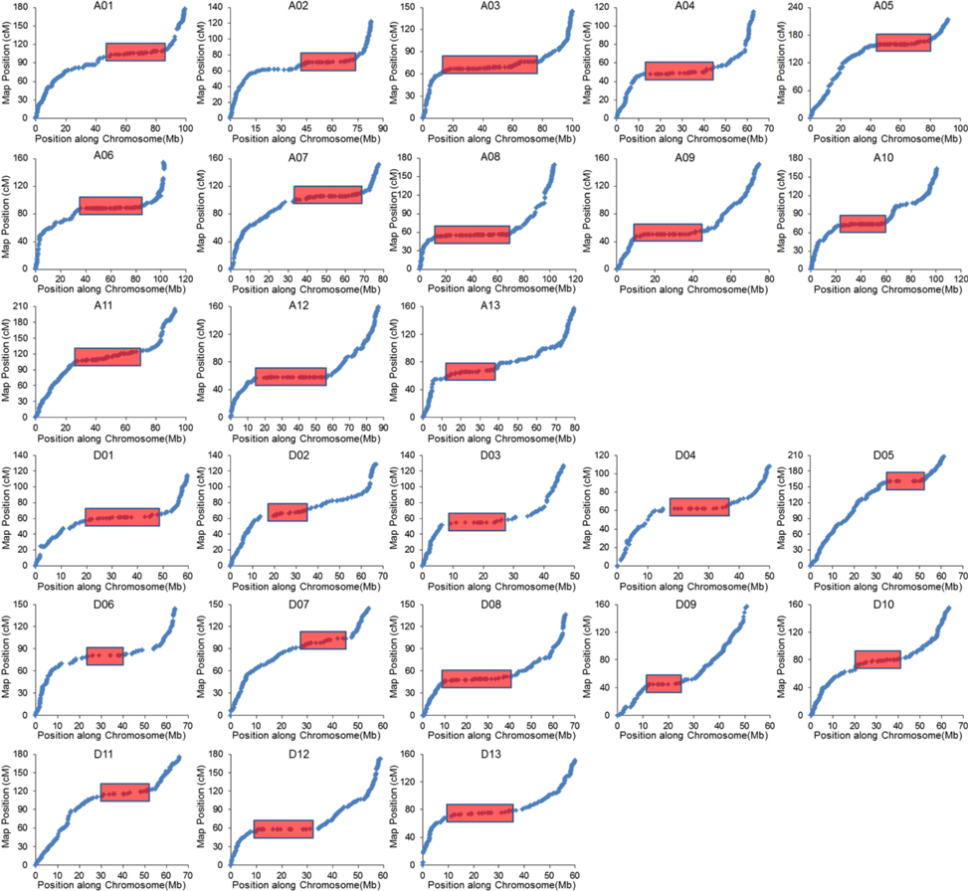
The marker placements for the genetic map on the *G. hirsutum* acc. TM-1 chromosomes. The marker order on the y-axis is derived from the genetic maps and the marker order on the x-axis is derived from the physical maps. Both the relative genetic and physical distances of the chromosomes on the plots are represented by the cells of different sizes according to the ratio of the chromosome lengths. Cumulative genetic distance in cM and physical distance in bp are indicated on x-axis and y-axis, respectively. The red rectangles represent the recombination suppression regions (nearly flat shaded) which are predicted to be pericentromeric regions

To facilitate genetic analysis, we converted the SNP linkage map into a skeleton bin map (Fig. 4). The two subgenomes had similar numbers of recombination bins in the skeleton bin map: 2,096 in the A subgenome and 1,953 in the D subgenome, making a total of 4,049. The average physical length of the recombination bins was 570 kb. The genetic and physical distances were not directly proportional, as shown in Fig. 5, where the plots of recombination bin placements on the 26 chromosomes are compared.

Recombination can generate genetic variation and induce genetic diversity. By comparing genetic and physical distances between adjacent markers, we further examined the relative changes in recombination rate along each chromosome. Consistently high recombination at chromosome ends and recombination suppression were clearly observed in different linkage groups. We found 26 recombination suppression regions in the 26 linkage groups (Fig. 5), which might be related to the heterochromatic regions [56]. These covered the repeat-rich centromeric or pericentromeric regions on each chromosome. We found that the recombination rates in hotspot regions were in the range of 1.4 to 5.7 cM/Mb, while in the suppression regions, recombination rates were in the range of 0 to 0.5 cM/Mb. Previous reports have revealed even more extreme variation in wheat [57, 58], where 1 Mb corresponds to a range of 0.05 to 8.47 cM, and in chromosome 4 of *Arabidopsis* [59], where 1 Mb corresponds to a range of 0 cM near the centromere to 20 cM on the short arm of the chromosome. Each chromosome was approximately subdivided into recombination hotspots (recombination-rich) and recombination suppression (recombination-poor) regions. The average ratio of genetic-to-physical distance was 1.75 cM/Mb for the whole genome of *G. hirsutum*. Genome-wide patterns of recombination have been described previously for some species, including *Arabidopsis* [59, 60] and rice [61]. This ratio is similar to those for watermelon (2.3 cM/Mb) [6], sorghum (1.43 cM/Mb) [62], and cucumber (3.2 cM/Mb) [63], but is lower than that for maize (5.5 cM/Mb) [64].

### Genetic mapping of centromeric regions in allotetraploid cotton chromosomes

Little is known of the dynamics of centromeric DNA in polyploidy plants. Centromeres usually contain high-copy-number retrotransposons and satellite repeats, which are difficult to map, clone, and sequence. Few studies have focused on centromeres in cotton [24, 65]. In our previous study, we found that BAC 97G20 hybridized to the centromeric regions of all 52 chromosomes in domesticated Upland cotton [66]. Sequence analysis of BAC 97G20 showed that there were four long terminal repeat (LTR) retrotransposons, named GhCR1-4, with a strong similarity to the centromeric retrotransposons reported in other plants [24]. Of them, GhCR1 and GhCR3 had 93.6 % and 94.4 % similarity to centromere retroelements in *Gossypium* (CRG), CRG1 and CRG2, respectively. CRG1 and CRG2 were recently identified by co-localization immunostaining with antiserum to the centromere-specific histone CENH3 in cotton [65], indicating that GhCR1 and GhCR3 may belong to the same class as the CRGs.

**Fig. 6:**
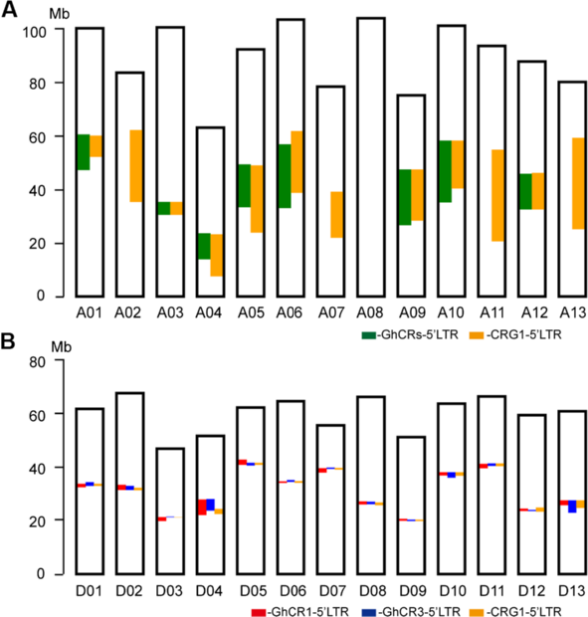
Schematic diagrams of centromeric regions of *G. hirsutum* acc. TM-1. **a** Centromeric regions of the A subgenome were identified by GhCRs-5’LTR (green) and CRG1-5’LTR (orange). **b** Centromeric regions of the D subgenome were identified by GhCR1-5’LTR (red), GhCR3-5’LTR (blue), and CRG1-5’LTR (orange)

Whole-genome sequence screening for four GhCR LTR homologies in *G. hirsutum* acc. TM-1 [41] identified 3,515 LTR homologies (about 270 for each chromosome on average) in the D subgenome; a much higher number than the 430 (about 33 for each chromosome on average) in the A subgenome (*T*-test: *P* value = 6.81e-07) (Additional file 9). This observation is consistent with the results of our FISH hybridization, which revealed that four of these retrotransposons were hybridized specifically to the centromere region in all of the chromosomes of *G. hirsutum*; however, much weaker signals were observed in the A subgenome than in the D subgenome. Of these retrotransposons, GhCR1 and GhCR3 LTRs had 1,857 and 1,375 homologous sequences, respectively; much higher than the 146 and 137 found in GhCR2 and GhCR4, respectively, in the TM-1 genome (Additional file 9). The putative centromeric regions in allotetraploid Upland cotton chromosomes are illustrated in Fig. 6. In the D subgenome, the centromeric regions were highly overlapped by GhCR1-5’LTR and GhCR3-5’LTR. With the exception of the predicted centromeric regions, which covered more than 4 Mb in D04 and D13, there were less than 2 Mb of centromeric regions in all other chromosomes (Fig. 6 and Additional file 10). In the A subgenome, however, no putative centromeric region was detected in A02, A07, A08, A11, or A13, with GhCRs absent in these chromosomes. In the other eight chromosomes, there was an extremely low level of homology with the predicted centromeric region sequences and a relatively large physical distance, ranging from 4.8 to 23.7 Mb, was detected; implying that there are large centromeric regions in the A subgenome (Fig. 6 and Additional file 10). In other plant species, the size of centromeric regions ranges from 9 Mb, in chromosome 1 of *Arabidopsis*, to 124 kb, in chromosome 4 of rice [67–70]. Although the sequences of centromeric regions show baffling diversification and species-specificity, the present report implies that the cotton centromeric regions span several hundred kilobases to several megabases. The centromeric regions identified in the study will serve as useful landmarks for fine-tuning the structure of the cotton genome.

**Fig. 7:**
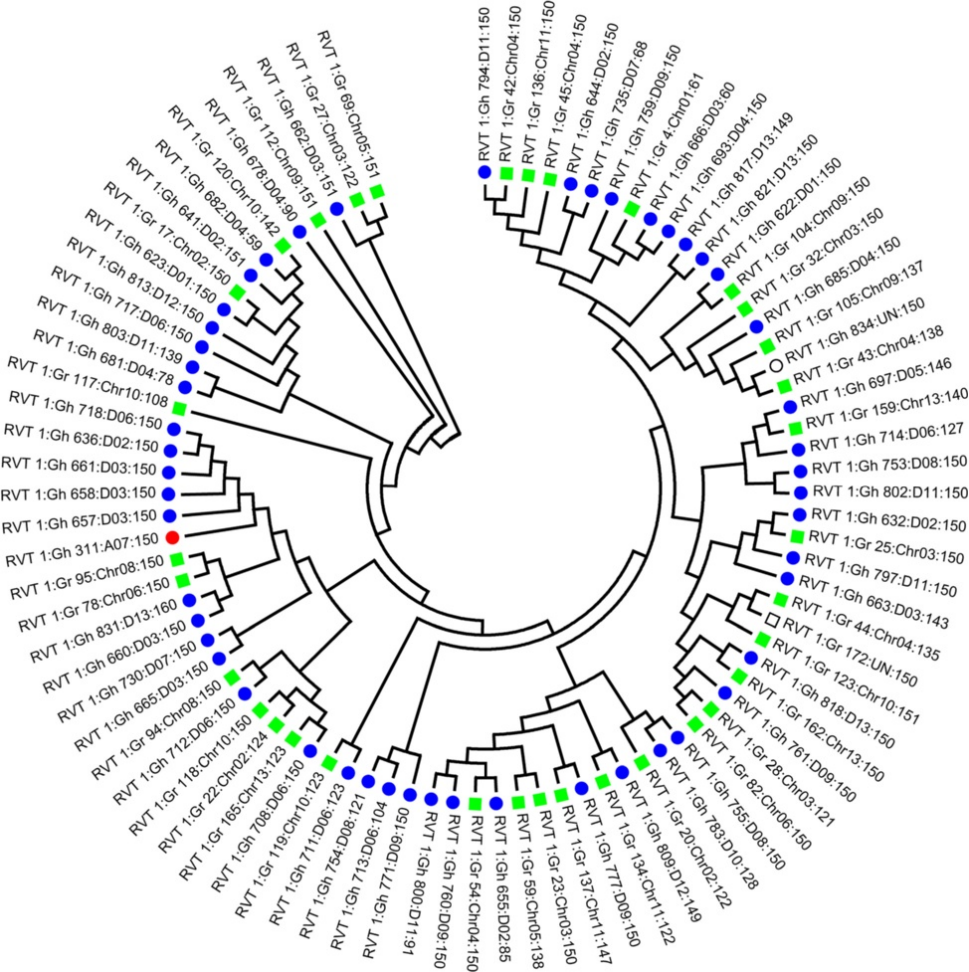
Neighbor-joining (NJ) phylogenetic tree based on RT amino acid sequences of GhCRs LTR from *G. raimondii* and *G. hirsutum*. Statistical support was evaluated by bootstrapping (1,000 replications); Green squares represent RTs from *G. raimondii*. Red and blue circles represent RTs from the A subgenome and D subgenome of TM-1, respectively. Hollow squares and circles represent RTs from scaffolds that were unanchored to chromosomes of *G. raimondii* and *G. hirsutum*, respectively

In recent years, the study of centromeres has progressed rapidly in cotton [24, 65]. FISH analysis revealed that CRGs [65] and GhCRs [24] are both found in all 52 chromosomes in allotetraploid cottons and in D genome diploid cotton species, but not in A genome diploid species, such as *G. arboreum* and *G. herbaceum*. Whole-genome screening of the *G. arboreum* genome sequence [47] found only 17 homologies of CRGs (sequence identity >80 %) [65] and 119 GhCR LTRs in the A genome. Furthermore, the number of CRG homologies [65] and GhCR LTRs are much higher in the D subgenome than in the A subgenome in allotetraploid cotton. No reverse transcriptase (RT) domain from GhCRs LTR retrotransposon was found in the diploid A genome. However, one RT domain of GhCRs LRT was found in the A subgenome of TM-1. Phylogenetic analysis was performed using the Neighbor-joining (NJ) method in the MEGA 5.10 program [71] and showed that this RT domain could be derived from the D genome/D subgenome (Fig. 7). Together, these results revealed that these centromeric retrotransposons may have been derived from the D subgenome progenitor, invaded the A subgenome centromeres in allotetraploid cotton during allopolyploid formation, and were amplified during its evolutionary history. Extensive retrotransposon amplification must have occurred through the genome and, in particular, in the pericentromeric regions. Transposon activation following hybridization or polyploidy formation has been detected in some plants [65, 72, 73]. To limit genomic damage, plant hosts have evolved RNA interference-meditated mechanisms to tame their endogenous retroelements [74].

## Conclusions

In this study, we constructed the first ultra-dense genetic map composed of approximately 5 million NGS sequence-derived SNP markers in allotetraploid cotton. This map has played an important role in genome assembly, the detection of genome rearrangements, and the identification of centromeric regions of polyploid cotton. The methodology used in this study can be applied to whole genome sequencing and the assembly of many other polyploid crops with large and complex genomes. Further, this complete and high resolution composite map is of practical importance for novel QTL discovery, gene mapping, map-based cloning, and marker assisted selection in cotton.

## Materials and methods

### Plant materials, DNA extraction, PCR amplification, and electrophoresis

The plant materials used consisted of *G. hirsutum* acc TM-1 and *G. barbadense* cv. Hai7124, two highly homozygous parents, as well as 59 F_2_ individuals derived from a cross between TM-1 as the recipient and Hai7124 as the donor parent. TM-1 is a genetic standard line of *G. hirsutum* developed through single plant selection.*barbadense* cv. Hai7124, grown extensively in China, was also a single plant selection offspring before it was used as a parent in the construction of the linkage map [75]. Genomic DNA were extracted from young leaf tissues following the methods described by Paterson *et al.* [76], with increased RNase A and proteinase K treatment to prevent RNA and protein contamination, before the extracts were subjected to Illumina sequencing technology and SSR-PCR amplification.

We selected 441 framework SSR markers from a complete linkage map [33], including 3,147 markers with 10-cM intervals between markers on 26 chromosomes, to anchor our framework map. Four hundred and forty-one SSR markers were employed to survey the 59 individuals in the F_2_ mapping population. SSR-PCR amplification and electrophoresis followed the methods described by Zhang *et al.* [75].

### Library construction and sequencing

Genomic libraries were prepared following the manufacturer’s standard instructions for high-throughput DNA sequencing for subsequent cluster generation, and were sequenced on the Illumina HiSeq 2000 platform (Illumina, San Diego, CA, USA). To construct paired-end libraries, DNA was fragmented by sonication, and DNA ends were blunted before adding an A base to each 3′ end. DNA adaptors with a single T-base 3′ end overhang were ligated to the above products. Ligation products were purified on 2 % agarose gels that each targeted a specific range of insert sizes. Quantification and quality assessment were carried out by running 1 μL of library on an Agilent DNA 1000 LabChip analyzer (Agilent Technology 2100 Bioanalyzer). We constructed *G. hirsutum* genome sequencing libraries with insert sizes of 180 bp and 300 bp, *G. barbadense* with 300 bp inserts, and each F_2_ individual with 300 bp inserts.

### Data Set extraction

Base-calling files were obtained from raw fluorescent images from the Illumina HiSeq 2000 platform using CASAVA 1.8 software in FASTQ format [77]. Illumina reads were filtered using NGSQCTookit v2.3 [78] (parameters of -cutOffReadLen4HQ 70, −cutOffQualScore 20).

### Sequence alignment to the temporary reference genome, SNP discovery, and genotyping

Alignment of the sequences to the TM-1 reference genome [41] was performed with BWA [37] using only sequences aligning to the reference genome with less than two mismatches based on a high quality sequence. Only sequences with a mapping score of at least 20 were used for SNP discovery.

For each parental sample, putative SNPs with respect to the TM-1 reference sequences were first called using samtools [38] and an in-house Perl script under the following conditions: minimum read depth at a given position was 10, minimum supporting reads for an allele was 3, and the allele represented at least 20 % of all the alleles observed. The interspecific SNPs (allelic polymorphisms), which can be broadly classified as simple SNPs, hemi-SNPs, and complex SNPs, were obtained from the two parental ‘putative SNPs’ lists using an in-house Perl script. Simple SNP types represent instances of either the reference sequence being genome-specific or sequence divergence of one subgenome so as to prevent alignment of one homoeolog to the reference sequence. Hemi-SNP types represent instances of reads from both subgenomes aligned to reference sequences with an allelic variation being present in one. Complex SNP types represent instances of an allelic variation having occurred at the site of an inter-homoeolog variation between the subgenomes. The plain-text format ‘pileup’ files encapsulating the alignment maps generated by samtools were inspected sequentially for each population sample over each of the SNP positions present in the SNP superset.

Using the mapping population, the initial genotypes of individuals were detected and classified into blocks based on similarity scores. The similarity score was calculated at SNP sites independently according to the following formula:

$$ {S}_z\kern0.5em =\kern0.5em \underset{i\kern0.5em =\kern0.5em 1}{\overset{n}{\varSigma }}\kern0.5em s\left({G}_{iz}{G}_{i\mathrm{j}}\right)\kern0.5em /\kern0.5em n $$

where *G*_*i*j_ and *G*_*i*z_ is the genotype of individuals *i* at the *j*-th SNP and at the *z*-th SNP, respectively, and *s*(*G*_*i*z_*G*_*i*j_) / *n* is the score of individuals *i* between the genotype at the *z*-th SNP and the genotype at the *j*-th SNP. *n* represents the number of individuals without the missing genotype at the *j* and *z*-th SNP. The similarity score is the sum of the scores of *n* individuals at the *z*-th SNP except for those with missing genotypes. The score of individual *i* at the *j* and *z*-th SNPs are: *s =* 1 if the genotypes of *G*_*i*z_ and *G*_*i*j_*G*_*i*j_ are identical (A vs. A, B vs. B, H vs. H); *s =* 0.5 if the genotypes of *G*_*i*z_ and *G*_*i*j_ are different and one is ‘H’ (A vs. H, B vs. H); and *s =* 0 if the genotypes of *G*_*i*z_ and *G*_*i*j_ are different and neither is ‘H’ (A vs. B). The minimum similarity score used for classifying similar SNP sites into a block was 0.7. There were two reasons that a minimum similarity score of 0.7 was used: (1) Markers with a high proportion of matches (>27/37, 72.97 %) were classified into a block in a similar study [5]; and (2) the accuracy of initial genotype calling at SNP sites for the F_2_ population was 80.07 %.

For SNP sites in the same scaffold and block, an approach combining the sliding window approach [79] and Bayesian inference [80] based on reads in the window was used to determine the genotypes of individuals that could reduce the error rate and the number of missing genotypes. For a genotype at a given *w* window, assume there are *n* reads at the SNP sites in the window. The error rate of parent 1 and parent 2 are *E*_1_ (0.058) and *E*_2_ (0.02), respectively. Under the condition of the allele from parent 1 (*P*_1_*P*_1_) or parent 2 (*P*_2_*P*_2_) or two parents (*P*_1_*P*_2_), the probability of observing n_1_ reads of parent 1 at the SNP sites in the window would follow a binominal distribution:

$$ \begin{array}{l}P\left({n}_1\kern0.5em \Big|\kern0.5em {P}_1{P}_1\right)\kern0.5em =\kern0.5em \left(\begin{array}{c}\hfill {n}_1\hfill \\ {}\hfill n\hfill \end{array}\right){\left(1\kern0.5em -\kern0.5em {E}_1\right)}^{n_1}\kern0.5em {E_2}^{n-{n}_1}\\ {}P\left({n}_1\kern0.5em \Big|\kern0.5em {P}_2{P}_2\right)\kern0.5em =\kern0.5em \left(\begin{array}{c}\hfill n\kern0.5em \mathit{\hbox{-}}\kern0.5em {n}_1\hfill \\ {}\hfill n\hfill \end{array}\right){E_1}^{n_1}{\left(1\kern0.5em -\kern0.5em {E}_2\right)}^{n-{n}_1}\\ {}P\left({n}_1\kern0.5em \Big|\kern0.5em {P}_1{P}_2\right)\kern0.5em =\kern0.5em \left(\begin{array}{c}\hfill {n}_1\hfill \\ {}\hfill n\hfill \end{array}\right){\left(1\kern0.5em -\kern0.5em {E}_1\kern0.5em +\kern0.5em {E}_2\right)}^{n_1}\kern0.5em \left(1\kern0.5em +\kern0.5em {E}_1\kern0.5em -\kern0.5em {E}_2\right)n\kern0.5em -\kern0.5em {n}_1\end{array} $$

Let *P*(*P*_1_*P*_1_ | *n*_1_), *P*(*P*_2_*P*_2_ | *n*_1_), *P*(*P*_1_*P*_2_ | *n*_1_), be the posterior probabilities that the allele comes from parent 1, parent 2, or two parents given the observed *n*_1_ reads of parent 1 at the SNP sites in the window. These probabilities were calculated as:

$$ \begin{array}{l}P\left({P}_1{P}_1\kern0.5em \Big|\kern0.5em {n}_1\right)\kern0.5em =\kern0.5em \frac{P\left({n}_1\kern0.5em \Big|\kern0.5em {P}_1{P}_1\right)P\left({P}_1{P}_1\right)}{P\left({n}_1\right)}\\ {}P\left({P}_2{P}_2\kern0.5em \Big|\kern0.5em {n}_1\right)\kern0.5em =\kern0.5em \frac{P\left({n}_1\kern0.5em \Big|\kern0.5em {P}_2{P}_2\right)P\left({P}_2{P}_2\right)}{P\left({n}_1\right)}\\ {}P\left({P}_1{P}_2\kern0.5em \Big|\kern0.5em {n}_1\right)\kern0.5em =\kern0.5em \frac{P\left({n}_1\kern0.5em \Big|\kern0.5em {P}_1{P}_2\right)P\left({P}_1{P}_2\right)}{P\left({n}_1\right)}\end{array} $$

The genotype of the allele was determined based on the highest posterior probability at the SNP sites in the window:

$$ \max \kern0.5em \left\{P\left({P}_1{P}_1\kern0.5em \Big|\kern0.5em {n}_1\right),\kern0.5em P\left({P}_2{P}_2\kern0.5em \Big|\kern0.5em {n}_1\right),\kern0.5em P\left({P}_1{P}_2\kern0.5em \Big|\kern0.5em {n}_1\right)\right\} $$

The prior probabilities for genotypes *P*_1_*P*_1_, *P*_2_*P*_2_ and *P*_1_*P*_2_ used for the Bayesian probability calculation were estimated based on the theoretical probabilities for an F_2_ population, that is, *P*_1_*P*_1_ : *P*_2_*P*_2_ : *P*_1_*P*_2_ = 0.25 : 0.25 : 0.5.

To evaluate the accuracy of the genotypes of the 59 individuals, the genotypes of 26 random SSR markers that can be uniquely mapped to scaffolds by the size of their PCR product were identified in each individual. We assumed that the SNP genotypes of the 59 individuals in 10 kb flank intervals of 26 SSR markers’ physical locations were consistent with the genotypes of the corresponding SSR markers.

### Genetic mapping and validation

After the imputed genotype was visually scored by a sliding window, unambiguous SNP sites were used for the construction of a genetic linkage map. Using SNP sites, an ultra-dense genetic linkage map based on the imputed genotype of allotetraploid cotton was constructed by the following procedure. The co-segregating SNP sites were clustered as a recombination bin, defined as a class of SNP sites that had identical genotypes across the 59 individuals with no recombination existing between each pair of SNP sites. The resulting 4,049 recombination bins combining SSR genotypes were used for the construction of the genetic linkage map using JoinMap Version 3.0 [39] with a recombination frequency <0.4 and minimum logarithm of odds (LOD) scores of 6. Recombination frequency was converted to linkage distances (centimorgan, cM) using the Kosambi mapping function. The resulting linkage groups constituted the genetic linkage map and were assigned the standard nomenclature for cotton (A01 to A13, D01 to D13) on the basis of the incorporation of a framework of SSR markers from the existing linkage map [33] and previously published chromosome naming systems [81]. To validate the linkage map, an independent program, CheckMatrix using PyMap BIT and REC scores, as described by Kozik [82], was utilized. The recombination bin map was used to anchor scaffolds and calculate recombination rates.

### Correcting the genome mis-assembly

Each scaffold within the SNP linkage map was divided into 201 bp segments based on the SNP positions. SNP segments were mapped to the assembled genome using BWA software [37]. The stable SNP segments were used to detect mis-assembly scaffolds and anchor scaffolds, which were identified if the following conditions were met: (1) the minimum match base was 201 bp for the TM-1 genome; (2) there was a unique alignment on the genome sequence; and (3) there were at least 10 consecutive SNP segments with the same genetic position.

### Integration of the newly generated genetic linkage map and the physical map of the *G. raimondii* genome

We aligned the scaffolds from the ultra-dense genetic linkage map based on the SNPs of allotetraploid cotton to the whole *G. raimondii* genome using MUMmer software, version 3.23 [83], and the best hit was chosen in the case of multiple matches.

### Estimation of recombination rates

The estimated recombination rate (cM/Mb) was calculated using in-house Perl scripts, which divided the genetic length of the segment in cM by the physical length of the segment in Mb. We defined recombination suppression regions as those where the recombination rate was less than 1.0 cM/Mb, and recombination hot regions as those where the recombination rate was greater than 1.0 cM/Mb [6].

### Linkage mapping of cotton centromeric regions

Four LTR retrotransposons were identified from the centromere-specific BAC clone, 97G20 [84], which is closely associated with the centromere of cotton. The retrotransposons were designated as GhCR1, GhCR2, GhCR3, and GhCR4, and more generally as GhCRs. By high-resolution mapping, the GhCRs were located in the centromeric regions of all 52 chromosomes in the tetraploid cotton, *G. hirsutum* acc TM-1. To search the centromeric regions of each chromosome in TM-1, a sequence alignment method was applied. According to the structure of the retrotransposons, relatively conserved 5’LTR regions, designated as GhCR1-5’LTR, GhCR2-5’LTR, GhCR3-5’LTR, and GhCR4-5’LTR, were selected as query sequences to align with the TM-1 genome database. All the alignments were obtained by BLASTn analysis with its parameters set to the default values. Subsequently, GhCRs-5’LTR were screened for sequence similarity and e-value, with ≥80 % and ≤1e-20 set as the threshold values, respectively. To identify the centromeric regions of the A subgenome, the screened alignments of 13 chromosomes from GhCRs-5’LTR were mingled. They were then used to calculate the median size of the 95 % confidence interval for the median, which was defined as the centromeric region for each chromosome. To identify the centromeric regions of the D subgenome, the screened alignments of 13 chromosomes from GhCR1-5’LTR and GhCR3-5’LTR were used to analyze the centromeric regions. For further analyses, we used a multiple alignment consensus sequence that contained the GhCRs LTR retrotransposon RT domain. Phylogenetic analysis was performed using the NJ method in the MEGA 5.10 program [71]. Statistical support for the NJ tree was evaluated by bootstrapping, where the number of replications was 1,000.

### Data access

The sequences have been deposited in DDBJ/EMBL/GenBank under the accessions (PRJNA274882). The SNP data between *G. hirsutum* acc TM-1 and *G. barbadense* cv. Hai7124 are available at http://mascotton.njau.edu.cn/. The F3 descended from F2 population will be available for mapping the QTL/genes related to important traits for predictive breeding in the near future.

## Additional files

Additional file 1:
**Processing workflow for assigning, ordering and orienting scaffolds and validating misassembled scaffolds.** First, sequencing the two parents (TM-1 and Hai7124) and an interspecific F_2_ population (THF_2_) derived from a cross between the two parents; second, tentatively assembling the WGS sequences of TM-1 using SOAPdenovo package [85]; third, SNP calling between two parents, genotyping, and constructing a high resolution SNP linkage map; fourth, validating the structural correctness of primarily assembled scaffolds of the TM-1 genome by the linkage map; fifth, further assembling the sequence scaffolds by integrating paired BAC-end sequences (BES) with default and stringent parameters, respectively; sixth, validating, anchoring and orienting of assembled scaffolds; finally, coupling construction of the ultra-dense genetic map for assembling and validating. Additional file 2:
**Information on reads mapped to the TM-1 reference sequence.**
Additional file 3:
**Distribution of HQ data in 59 individuals of (TM-1 × Hai7124)F**_**2**_ **.** To construct an ultra-dense linkage map in tetraploid cotton, population sequencing by low-depth (approximately 5× coverage of the genome) whole genome sequencing (WGS) of 59 F_2_ individuals was performed. The x axis indicates the 59 individuals of (TM-1 × Hai7124)F_2_. The y axis indicates the number of the high quality sequence reads (Gb). Additional file 4:
**Marker details for the ultra-dense SNP genetic linkage map and the corresponding framework SSRs linkage map.** Table listing the position of SSR markers in corresponding chromosomes, SSR marker name, and the genotypes of 59 F_2_ individuals for the framework SSRs linkage map, the position of the marker or scaffold in the corresponding chromosome, the ID of recombination bin, the region ID of the scaffold, the scaffold name, the length of the scaffold, the number of SNPs, the genotypes of the 59 F_2_ individuals, and the number of SNPs per recombination bin for the ultra-dense genetic linkage map. Alleles: A/A (red) = homozygous TM-1 genotype (maternal parent); B/B (blue) = homozygous Hai7124 genotype (paternal parent); H/H (yellow) = heterozygous genotype (TM-1 × Hai7124)F_1_; C = not genotype A (dominant B-allele); D = not genotype B (dominant A-allele); Missing data (genotype unknown) are noted as “—”. Additional file 5:
**Comparison of two genetic maps of inter-specific SNPs and intra-specific SNPs.** Intra-specific SNP tags in 104 linkage groups (purple) were aligned to TM-1 scaffolds in the 26 linkage groups based on inter-specific SNPs (blue). Additional file 6: Tables S1 and S2. Coordinates for splitting of the TM-1 genome assembly scaffolds. Tables S1 and S2, listing the misassembled scaffold name, the position of the misassembled scaffold mapped in different chromosomes, the breakout region of the misassembled scaffold, and the types of mis-assembly for the TM-1 genome initial assembly and version 1.0, respectively. Additional file 7:
**Genome variations identified via comparative mapping against D genome pseudomolecules.**
Additional file 8:
**SNP-poor regions between TM-1 and Hai7124.**
Additional file 9:
**Aligned** ***G. hirsutum*** **TM-1 genome with four sequences of GhCRs-5’LTR and CRG1-5’LTR.**
Additional file 10: Tables S1 and S2. Centromere mapping in the physical map of the TM-1 genome. Tables S1 and S2 listing centromere mapping in the physical map of the TM-1 A and D subgenomes, respectively.

## Abbreviations

BAC: Bacterial artificial chromosomes
bp: Base pair
BWA: Burrows-wheeler aligner
Chr: Chromosome
cM: centimorgan
CR: Centromeric retrotransposon
FISH: Fluorescent *in situ* hybridization
Gb: Gigabase
GBS: Genotyping-by-sequencing
kb: kilobase
LOD: Logarithms of odds
LTR: Long terminal repeat
Mb: Megabase
MQ: Mapping quality
NGS: Next-generation sequencing
PCR: Polymerase chain reaction
QTL: Quantitative trait loci
RAD: Restriction site-associated genomic DNA
SBG: Sequence-based genotyping
SNP: Single nucleotide polymorphism
SSR: Simple sequence repeats
WGS: Whole genome shotgun
