# Supplementary figures and images for: Sequence-based ultra-dense genetic and physical maps reveal structural variations of allopolyploid cotton genomes
Source: Genome Biol. 2015 May 24;16(1):108. doi: 10.1186/s13059-015-0678-1 (PMC4469577; doi:10.1186/s13059-015-0678-1)

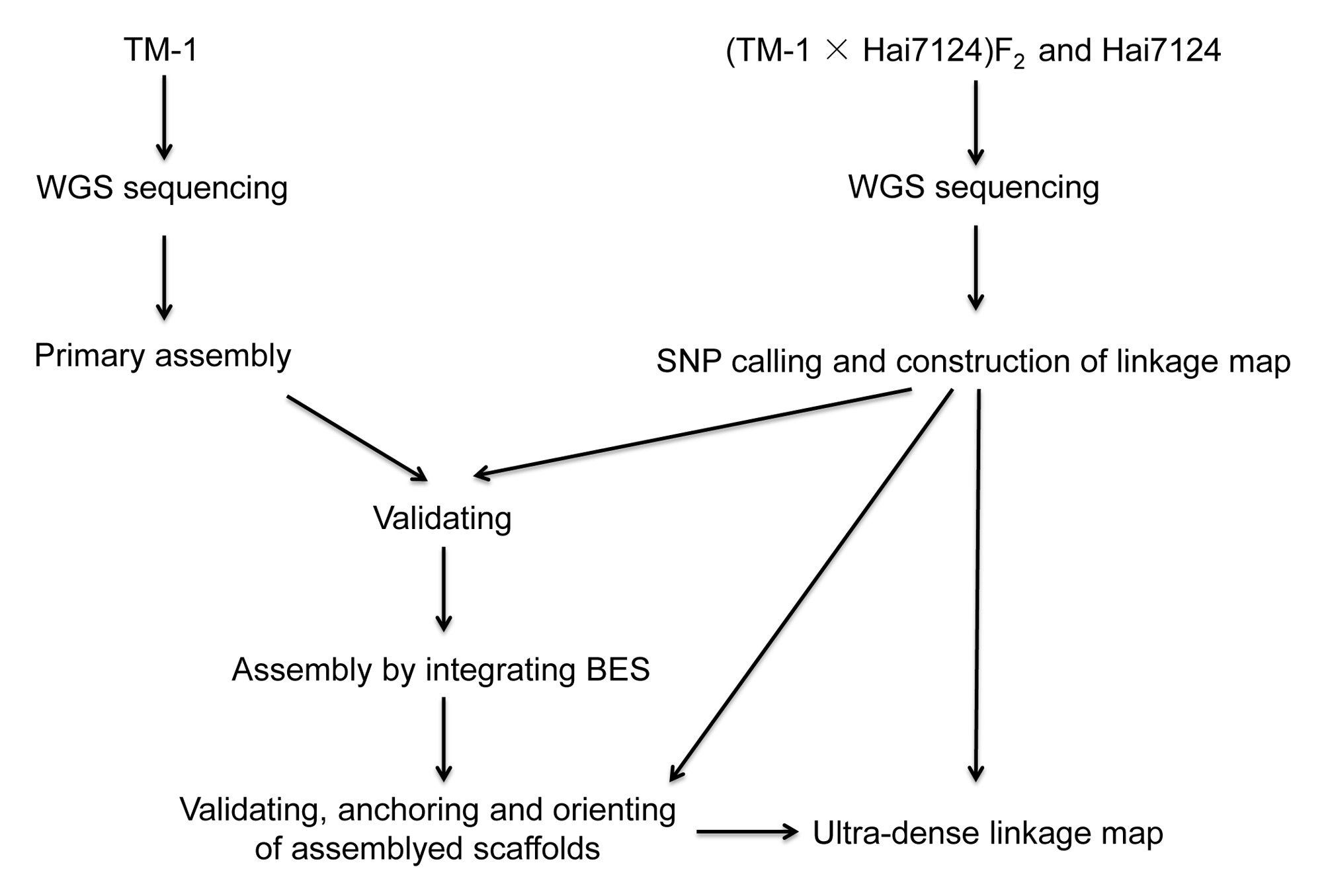

Supplement: Additional file 1: — Processing workflow for assigning, ordering and orienting scaffolds and validating misassembled scaffolds. First, sequencing the two parents (TM-1 and Hai7124) and an interspecific F2 population (THF2) derived from a cross between the two parents; second, tentatively assembling the WGS sequences of TM-1 using SOAPdenovo package [85]; third, SNP calling between two parents, genotyping, and constructing a high resolution SNP linkage map; fourth, validating the structural correctness of primarily assembled scaffolds of the TM-1 genome by the linkage map; fifth, further assembling the sequence scaffolds by integrating paired BAC-end sequences (BES) with default and stringent parameters, respectively; sixth, validating, anchoring and orienting of assembled scaffolds; finally, coupling construction of the ultra-dense genetic map for assembling and validating. [file 13059_2015_678_MOESM1_ESM.tif]

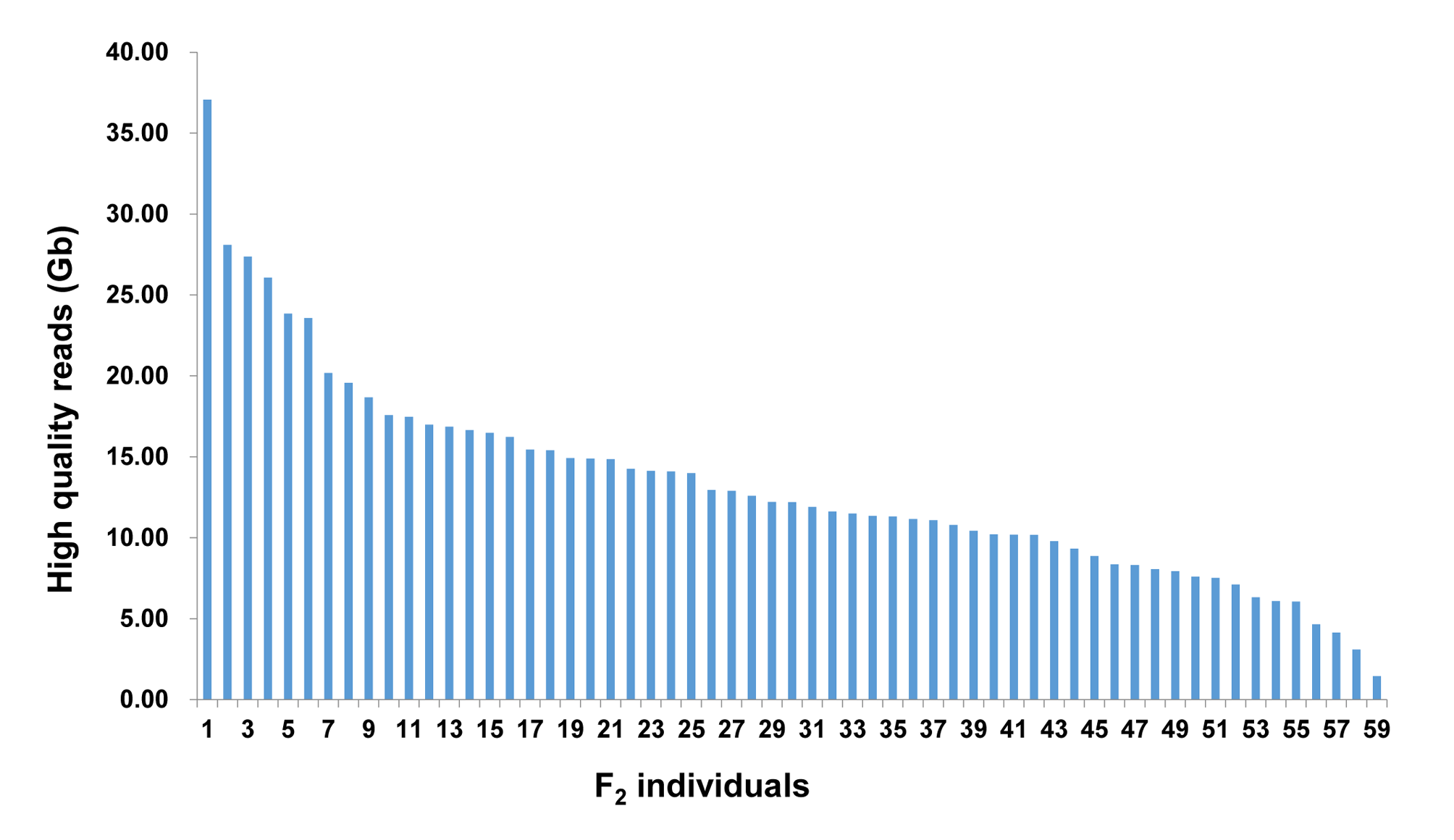

Supplement: Additional file 3: — Distribution of HQ data in 59 individuals of (TM-1 × Hai7124)F 2 . To construct an ultra-dense linkage map in tetraploid cotton, population sequencing by low-depth (approximately 5× coverage of the genome) whole genome sequencing (WGS) of 59 F2 individuals was performed. The x axis indicates the 59 individuals of (TM-1 × Hai7124)F2. The y axis indicates the number of the high quality sequence reads (Gb). [file 13059_2015_678_MOESM3_ESM.tif]

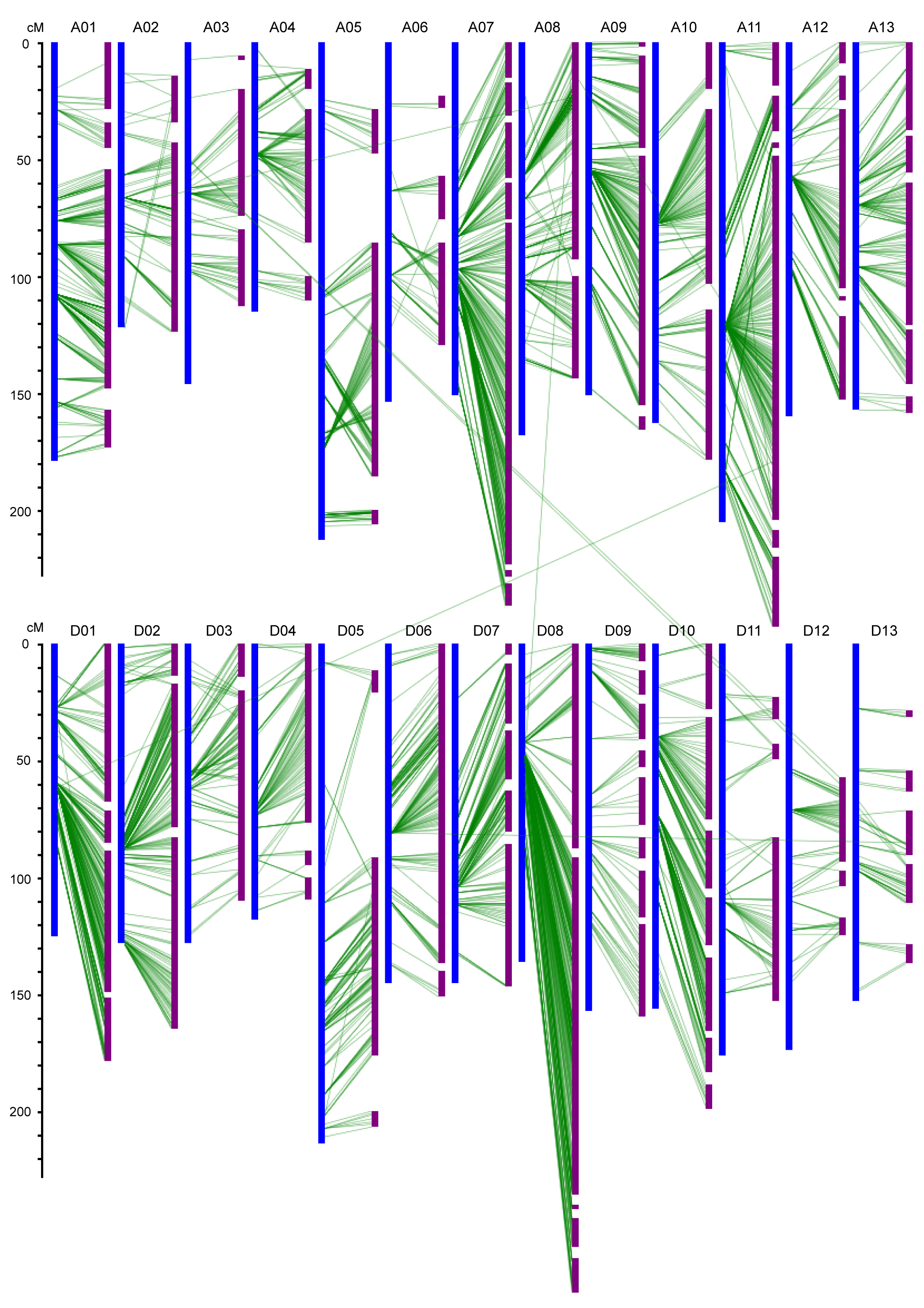

Supplement: Additional file 5: — Comparison of two genetic maps of inter-specific SNPs and intra-specific SNPs. Intra-specific SNP tags in 104 linkage groups (purple) were aligned to TM-1 scaffolds in the 26 linkage groups based on inter-specific SNPs (blue). [file 13059_2015_678_MOESM5_ESM.tif]
