# Supplementary material for: Sequence-based ultra-dense genetic and physical maps reveal structural variations of allopolyploid cotton genomes
Source: Genome Biol. 2015 May 24;16(1):108. doi: 10.1186/s13059-015-0678-1 (PMC4469577; doi:10.1186/s13059-015-0678-1)
Supplement: Additional file 2: — Information on reads mapped to the TM-1 reference sequence. [file 13059_2015_678_MOESM2_ESM.doc]

**Additional file 2. The information of reads mapped to TM-1 reference sequence.**

|  | **Unique alignment reads** | | **Multi-alignment reads** | **Unalignment reads** |
| --- | --- | --- | --- | --- |
|  | **MQ >20** | **MQ ≤20** |
| TM-1 | 1,238,953,773 (72.54 %) | 69,011,739 (4.04 %) | 308,811,971 (18.08 %) | 91,270,777 (5.34 %) |
| Hai7124 | 406,003,831 (38.69 %) | 389,070,897 (37.08 %) | 188,877,054 (18.00 %) | 65,410,974 (6.23 %) |
