# Supplementary material for: Sequence-based ultra-dense genetic and physical maps reveal structural variations of allopolyploid cotton genomes
Source: Genome Biol. 2015 May 24;16(1):108. doi: 10.1186/s13059-015-0678-1 (PMC4469577; doi:10.1186/s13059-015-0678-1)
Supplement: Additional file 8: — SNP-poor regions between TM-1 and Hai7124. [file 13059_2015_678_MOESM8_ESM.doc]

**Additional file 8. SNP-poor regions between TM-1 and Hai7124.**

| **ID** | **Chr.** | **Start (Mb)** | **End (Mb)** | **Length (Mb)** | **SNPs/50kb** |
| --- | --- | --- | --- | --- | --- |
| A01_1 | A01 | 41.85 | 45.2 | 3.35 | 3.58 |
| A01_2 | A01 | 45.5 | 75.05 | 29.55 | 2.47 |
| A01_3 | A01 | 86.85 | 89.95 | 3.1 | 3.07 |
| A01_4 | A01 | 91 | 92.3 | 1.3 | 2.32 |
| A05_1 | A05 | 13.05 | 14.75 | 1.7 | 8.76 |
| A06_1 | A06 | 18.15 | 21.85 | 3.7 | 8.57 |
| A08_1 | A08 | 73.7 | 80.05 | 6.35 | 9.33 |
| D04_1 | D04 | 9.4 | 13.15 | 3.75 | 7.74 |
| D11_1 | D11 | 10.45 | 11.65 | 1.2 | 3.62 |
