# Supplementary material for: Sequence-based ultra-dense genetic and physical maps reveal structural variations of allopolyploid cotton genomes
Source: Genome Biol. 2015 May 24;16(1):108. doi: 10.1186/s13059-015-0678-1 (PMC4469577; doi:10.1186/s13059-015-0678-1)
Supplement: Additional file 10: Tables S1 and S2. — Centromere mapping in the physical map of the TM-1 genome. Tables S1 and S2 listing centromere mapping in the physical map of the TM-1 A and D subgenomes, respectively. [file 13059_2015_678_MOESM10_ESM.doc]

**Additional file 10. Table S1. Centromere mapping in physical map of TM-1 A subgenome.**

| Chromosome | GhCRs-5’LTR | | |  | CRG1-5’LTR | | |
| --- | --- | --- | --- | --- | --- | --- | --- |
|  | Median (Mb) | 95 % CIM (Mb) | Size of 95 % CIM (Mb) |  | Median (Mb) | 95 % CIM (Mb) | Size of 95 % CIM (Mb) |
| A01 | 57.34 | 47.1-60.4 | 13.2 |  | 55.41 | 52.0-59.9 | 7.9 |
| A02 | - | - | - |  | 44.26 | 35.3-62.0 | 26.7 |
| A03 | 33.86 | 30.5-35.3 | 4.8 |  | 34.09 | 30.5-35.3 | 4.8 |
| A04 | 21.52 | 13.9-23.6 | 9.6 |  | 20.85 | 7.5-23.2 | 15.7 |
| A05 | 45.62 | 33.3-49.2 | 15.9 |  | 42.2 | 23.8-48.8 | 25.1 |
| A06 | 52.02 | 33.0-56.7 | 23.7 |  | 56.66 | 38.7-61.6 | 22.9 |
| A07 | - | - | - |  | 25.74 | 21.9-39.1 | 17.2 |
| A08 | - | - | - |  | - | - | - |
| A09 | 29.27 | 26.6-47.3 | 20.7 |  | 30.59 | 28.3-47.3 | 19 |
| A10 | 42.35 | 35.1-58.1 | 22.9 |  | 50.87 | 40.3-58.1 | 17.8 |
| A11 | - | - | - |  | 44.24 | 20.6-54.7 | 34.2 |
| A12 | 44.49 | 32.5-45.7 | 13.1 |  | 37.72 | 32.5-46.0 | 13.5 |
| A13 | - | - | - |  | 40.73 | 25.1-59.1 | 34 |

**Additional file 10. Table S2. Centromere mapping in physical map of TM-1 D subgenome.**

| Chromosome | GhCR1-5'LTR | | |  | GhCR3-5'LTR | | |  | CRG1-5'LTR | | |
| --- | --- | --- | --- | --- | --- | --- | --- | --- | --- | --- | --- |
|  | Median ( Mb ) | 95% CIM ( Mb ) | Size of 95% CIM ( Mb ) |  | Median ( Mb ) | 95% CIM ( Mb ) | Size of 95% CIM ( Mb ) |  | Median ( Mb ) | 95% CIM ( Mb ) | Size of 95% CIM ( Mb ) |
| D01 | 33.17 | 32.3-33.6 | 1.26 |  | 33.37 | 32.8-34.2 | 1.37 |  | 33.29 | 32.8-33.6 | 0.84 |
| D02 | 32.09 | 31.3-33.2 | 1.97 |  | 31.99 | 31.3-32.8 | 1.55 |  | 31.5 | 31.2-32.1 | 0.84 |
| D03 | 21.04 | 19.7-21.1 | 1.45 |  | 21.19 | 21.1-21.3 | 0.22 |  | 21.13 | 21.1-21.2 | 0.11 |
| D04 | 23.52 | 21.9-27.7 | 5.75 |  | 27.67 | 23.6-27.9 | 4.32 |  | 23.59 | 22.3-24.2 | 1.86 |
| D05 | 41.26 | 40.7-42.6 | 1.9 |  | 41.03 | 40.4-41.4 | 1.07 |  | 40.95 | 40.7-41.4 | 0.73 |
| D06 | 34.34 | 33.9-34.5 | 0.57 |  | 34.61 | 34.3-35.0 | 0.74 |  | 34.5 | 34.0-34.6 | 0.62 |
| D07 | 39.14 | 37.8-39.4 | 1.6 |  | 39.38 | 39.2-39.6 | 0.45 |  | 39.18 | 38.9-39.5 | 0.6 |
| D08 | 26.43 | 25.9-27.0 | 1.14 |  | 26.17 | 26.0-26.9 | 0.86 |  | 26.17 | 25.6-26.6 | 1 |
| D09 | 19.97 | 19.8-20.5 | 0.77 |  | 19.91 | 19.7-20.2 | 0.45 |  | 19.82 | 19.7-20.2 | 0.46 |
| D10 | 36.98 | 36.7-37.9 | 1.1 |  | 36.56 | 35.9-37.9 | 1.67 |  | 37.58 | 36.6-37.9 | 1.31 |
| D11 | 40.35 | 39.4-41.0 | 1.55 |  | 40.42 | 40.3-41.2 | 0.83 |  | 40.42 | 40.2-41.2 | 1.01 |
| D12 | 23.65 | 23.4-24.3 | 0.88 |  | 23.43 | 23.4-23.8 | 0.35 |  | 23.76 | 23.2-24.7 | 1.44 |
| D13 | 25.73 | 25.6-27.4 | 1.75 |  | 25.64 | 22.8-27.4 | 4.53 |  | 25.73 | 24.6-27.4 | 2.77 |
